# Understanding Sensitization, Cognitive and Neuropathic Associated Mechanisms behind Post-COVID Pain: A Network Analysis

**DOI:** 10.3390/diagnostics12071538

**Published:** 2022-06-24

**Authors:** César Fernández-de-las-Peñas, Manuel Herrero-Montes, Ignacio Cancela-Cilleruelo, Jorge Rodríguez-Jiménez, Paula Parás-Bravo, Umut Varol, Pablo del-Valle-Loarte, Gema Flox-Benítez, Lars Arendt-Nielsen, Juan A. Valera-Calero

**Affiliations:** 1 Department of Physical Therapy, Occupational Therapy, Rehabilitation and Physical Medicine, Universidad Rey Juan Carlos, 28922 Alcorcón, Spain; ignacio.cancela@urjc.es (I.C.-C.); jorge.rodriguez@urjc.es (J.R.-J.); 2 Center for Neuroplasticity and Pain (CNAP), SMI, Department of Health Science and Technology, Faculty of Medicine, Aalborg University, DK-9220 Aalborg, Denmark; lan@hst.aau.dk; 3 Departamento de Enfermería, Universidad de Cantabria, 39008 Santander, Spain; manuel.herrero@unican.es (M.H.-M.); paula.paras@unican.es (P.P.-B.); 4 Grupo de Investigación en Enfermería, Instituto de Investigación Sanitaria Valdecilla (IDIVAL), 39011 Santander, Spain; 5 VALTRADOFI Research Group, Department of Physiotherapy, Faculty of Health, Camilo Jose Cela University, 28692 Villanueva de la Cañada, Spain; umut.varol@alumni.ie.edu; 6 Department of Internal Medicine, Hospital Universitario Severo Ochoa, 28911 Leganes, Spain; pablo.valle@salud.madrid.org (P.d.-V.-L.); gema.flox@salud.madrid.org (G.F.-B.); 7 Department of Medical Gastroenterology, Mech-Sense, Aalborg University Hospital, DK-9000 Aalborg, Denmark; 8 Department of Physiotherapy, Faculty of Health, Universidad Camilo José Cela, 28692 Villanueva de la Cañada, Spain

**Keywords:** COVID-19, pain, post-COVID, network, neuropathic, sensitization, anxiety

## Abstract

This study aimed to describe a network including demographic, sensory-related, psychological/cognitive and other variables in individuals with post-COVID pain after hospitalization. Demographic (i.e., age, height, weight, months with symptoms), sensory-related (Central Sensitization Inventory -CSI-, Self-Report Leeds Assessment of Neuropathic Symptoms -S-LANSS-, PainDETECT), psychological/cognitive (Hospital Anxiety and Depression Scale -HADS-A/HADS-D-, Pain Catastrophizing Scale -PCS-, Tampa Scale for Kinesiophobia -TSK-11-) and other (sleep quality and health-related quality of life -EQ/5D/5L) variables were collected in 146 COVID-19 survivors with post-COVID pain. A network analysis was conducted to quantify the adjusted correlations between the modelled variables, and to assess their centrality indices (i.e., the connectivity with other symptoms in the network and the importance in the system modelled as network). The network revealed associations between sensory-related and psychological/cognitive variables. PainDETECT was associated with S-LANSS (ρ: 0.388) and CSI (ρ: 0.207). Further, CSI was associated with HADS-A (ρ: 0.269), TSK-11 (ρ: 0.165) and female gender (ρ: 0.413). As expected, HADS-A was associated with HADS-D (ρ: 0.598) and TSK-11 with PCS (ρ: 0.405). The only negative association was between sleep quality and EQ-5D-5L (ρ: −0.162). Gender was the node showing the highest strength, closeness, and betweenness centralities. In addition, CSI was the node with the second highest closeness and betweenness centralities, whereas HADS-D was the node with the second highest strength centrality. This is the first study applying a network analysis for phenotyping post-COVID pain. Our findings support a model where sensitization-associated symptoms, neuropathic phenotype, and psychological aspects are connected, reflecting post-COVID pain as a nociplastic pain condition. In addition, post-COVID pain is gender dependent since female sex plays a relevant role. Clinical implications of current findings, e.g., developing treatments targeting these mechanisms, are discussed.

## 1. Introduction

Evidence supports the presence of multiple symptoms after the acute phase of Severe Acute Respiratory Syndrome Coronavirus-2 (SARS-CoV-2) infection, the agent causing coronavirus disease 2019 (COVID-19), in up to 60% of patients [1,2]. The presence of post-COVID symptoms is called long COVID [3]. Fatigue and dyspnea are the most prevalent post-COVID symptoms [1,2]; although pain is likewise a highly prevalent symptom [4]. Increasing evidence supports that post-COVID pain resembles musculoskeletal features [5]; but around 20% of patients with post-COVID pain also exhibit neuropathic pain [6].

Phetopying of post-COVID pain can be crucial for better understanding of potential mechanisms and for orientating personalized-treatment based on mechanism phenotypes. Musculoskeletal chronic pain, but also neuropathic pain conditions can be associated with sensitization-associated symptoms, the underlying concept for defining “nociplastic pain” [7]. Nociplastic pain is defined as “pain that arises from altered nociception without clear evidence of actual or threatened tissue damage causing the activation of peripheral nociceptors or evidence for disease or lesion of the somatosensory system causing pain” [8]. Nociplastic pain conditions are not just associated with exaggerated pain responses, but also with central nervous system-derived symptoms such as fatigue, sleep problems, memory loss, and psychological disturbances [9]. All these features have been observed in individuals with long COVID since fatigue and memory loss are highly prevalent post-COVID symptoms [1,2]. Further, mood disorders and sleep problems [10] or sensitization-associated symptoms [11] have also been identified in people with long COVID.

A theoretical framework of long COVID considers reciprocal interaction between biology and behaviours, accordingly application of network analyses could help to better understand the interactions between sensory, cognitive, and psychological variables in individuals developing post-COVID pain. Network analysis provides a methodology to understand complex relationships [12] and is able to identify the most important variables (nodes) in an identified network [13]. From a network perspective, post-COVID pain may be sustained by multiple complex interactions between clinical, psychological, cognitive, and physiological systems. In such s scenarion, network analysis has been previously used to better understand the complexity of different pain conditions of musculoskeletal [14] or neuropatic [15] origin. No previous study has used network analysis for phenotyping post-COVID pain.

The main aim of the current study was to describe a network including demographic, sensory-related, psychological, and other variables in individuals with post-COVID pain. We also illustrate the potential of a network analysis for understanding the associations of different aspects of post-COVID pain by generating research questions, and improving treatment strategies.

## 2. Methods

### 2.1. Participants

This cohort study included subjects who had been previously hospitalized because SARS-CoV-2 infection in three urban hospitals in Spain. The diagnosis was conducted with real-time reverse transcription-polymerase chain reaction assay of nasopharyngeal and/or oral swab samples and the presence of clinical/radiological findings at hospital admission. Patients were included if presented “de novo” pain symptoms for at least three months starting after the acute infection (hospital discharge) and the absence of any underlying medical condition which could explain pain, e.g., arthritis. They were excluded if reported history of pain before the infection and any existing medical comorbidity explaining pain symptomatology to reduce confounding variables prior to hospitalization. Participants were scheduled for a face-to-face interview at follow-up period longer than one year after hospital discharge. This study was approved by the Local Institutional Ethics Committees (INDIVAL Cantabria 2020.416; HUIL/092-20, HUFA 20/126; URJC0907202015920; HSO 25112020).

### 2.2. Sensory-Related Variables

Neuropathic and sensitization-associated symptoms were assessed with validated patient-reported outcome measures (PROM). We used the Self-Report Leeds Assessment of Neuropathic Symptoms (S-LANSS) [16] and the PainDETECT [17] for evaluating the neuropathic component; and the Central Sensitization Inventory (CSI) [18] for assessing the presence of sensitization-associated symptoms.

The S-LANSS uses a binary response where subjects confirm whether they experience of different symptoms to classify them into a predominantly or non-predominantly neuropathic origin [16]. The Spanish version of the S-LANSS has shown good sensitivity, internal consistency and validity [16]. The S-LANSS score ranges from 0 to 24 points. A score ≥ 12 points suggests that the subject is susceptible of neuropathic pain symptoms [16].

The Spanish version of the PainDETECT questionnaire has shown high sensitivity (85%), specificity (80%), and and a positive predictive accuracy (83%) for identifying neuropathic pain [17]. It includes 9 items (seven pain-symptom items, one pain-course, and one pain-irradiation). The total score ranges from 0 to 38 points and uses the following cut-off values: <12 points, unlikely neuropathic component; 12–18 points, ambiguous neuropathic component; or >18 points, likely neuropathic component [17].

The CSI includes 25 health-related symptoms associated to sensitization into a 5-point Likert scale and it has been adapted to Spanish language [18]. Its score ranges from 0 to 100 points, where >40 points suggest the presence of sensitization-associated symptoms [19]. It has shown psychometric strength for assessing symptoms of sensitization in patients with persistent pain [20].

### 2.3. Psychological Variables

The Spanish version of the Hospital Anxiety and Depression Scale (HADS) was used to evaluate anxiety and depressive levels [21]. We included anxiety (HADS-A, 7-items, 0–21 points) and depression (HADS-D, 7-items, 0–21 points) scales. Higher scores suggest higher levels of anxiety or depressive symptoms [21]. We considered the cut-off scores recommended for the Spanish population indicative of anxiety (HADS-A ≥ 12 points) or depressive (HADS-D ≥ 10 points) symptoms [22].

Pain catastrophizing was evaluated with the Spanish version of the Pain Catastrophizing Scale (PCS) [23]. The PCS consists of 13-items evaluating rumination, magnification, and despair aspects in relation to pain symptoms. Items are answered in a 5-point Likert scale where 0 means “never” and 4 means “always” (total score 0–52) [23].

Fear of movement, also known as kinesiophobia, was assessed with Spanish version of the 11-items Tampa Scale Kinesiophobia (TSK-11) [24]. The TSK-11 includes 11 questions where the patients had to choose how much they agree or disagree with each item, being 1 “complete disagreement” and 4 “complete agreement” (score from 0 to 44) [24].

### 2.4. Health-Related Quality of Life

Health-related quality of life was assessed with the paper-based five-level version of EuroQol-5D questionnaire [25]. The EuroQol-5D-5L evaluates mobility, self-care, daily activities, pain and depression/anxiety dimensions from 0 (no problems) to 3 (severe problems). Responses were converted into a single index number between 0 (death) and 1 (optimal health), by applying crosswalk index values for Spain life [26].

### 2.5. Sleep Quality

The Spanish version of the Pittsburgh Sleep Quality Index (PSQI) was used for evaluating the quality of sleep the previous month by asking topics regarding usual bedtime, wake-up time, number of hours slept, and time needed to fall asleep [27]. All questions are answered on a 4-point Likert scale (0 to 3), where greater scores suggest worse sleep quality (total score 0–21 points) [27].

### 2.6. Data Analysis

All analyses were conducted using the R software v.4.1.1. (RStudio, Boston, MA, USA) for Windows 10. In addition, the following libraries were installed and used for different purposes: qgraph (v.1.6.9) and glasso (v.1.11) for network estimation; and bootnet (v.1.4.3) for stability analysis.

This network was constituted by the following 14 variables set as nodes: age, weight, height, EuroQol-5D, PainDETECT, S-LANSS, CSI, HADS-A, HADS-D, PCS, TSK-11, post-COVID duration, and PSQI as continuous variables, and gender included as categorical. Edges in the network were represented by lines expressing the magnitude of the association by thickness ranging from 0 to 1. Direction of the partial correlations (ρ) are expressed as red colour for negative associations and green colour for positive associations. In addition, grey colour was used for those categorical variables where no sign is defined [28].

The network structure was determined based on the importance of each node based on strength, closeness, and betweenness centrality indices. Node strength is a blunt measure that takes node’s total level of involvement in the network and not the number of connections with other nodes, being clinically useful to determine which outcomes should be targeted for inducing direct changes in other variables. Closeness, defined as the inverse sum of the distances of shortest paths of the target node from all other nodes in the network, was interpreted as the expected speed of arrival of something flowing through the network. Therefore, targeting outcomes with high closeness could induce changes to other nodes more quickly than the nodes that are peripheral. Finally, betweenness centrality can be interpreted as the percentage of shortest paths that must go through the target node. Therefore, a node with a high betweenness centrality would act as an intermediary in the transmission of information or resources between other nodes or even clusters of nodes in the network [14].

Finally, edge weights and centrality indices variability were analyzed using a 1000 iterations bootstrapping (95% CI). The edge weights bootstrapped CIs were interpreted as accuracy of the estimated weights since only the edges with non-zero weights were preserved. For assessing the variability of the centrality indices (CS-coefficient as a measure of correlation stability), participant-dropping subset bootstrap was utilized. This method reflects the maximum proportion of data which could be dropped to retain >0.7 of the correlation with the original centrality indices [29].

## 3. Results

An initial sample of 200 patients with post-COVID symptoms were screened for participation. Fifty-four patients were excluded because their main post COVID symptom was fatigue or dyspnea, not pain. Accordingly, 146 (73%) fulfilled all criteria, agreed to participate and were evaluated a mean of 18.8 ± 1.8 months after hospital discharge. A preliminary exploratory data analysis revealed some missing values in the PainDETECT (*n* = 3) and S-LANSS (*n* = 3) questionnaires; accordingly, 8 records (2.01%) were dropped out from the dataset. Data from 141 patients were finally included in the analyses. Descriptive statistics of the variables used in the network can be found in Table 1.

**Figure 1:**
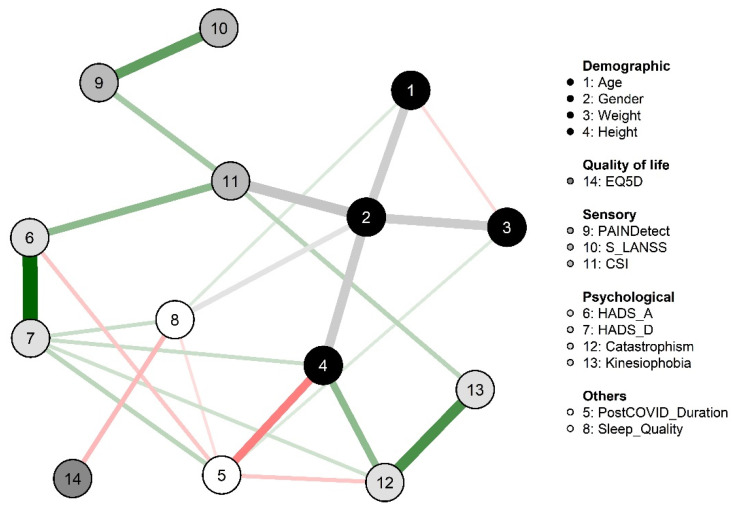
Network analysis of the association between demographic, sensory, psychological, quality of life and other measures in COVID-19 survivors with post-COVID pain. Edges represent connections between two nodes and are interpreted as the existence of an association between two nodes, adjusted for all other nodes. Each edge in the network represents either positive regularized adjusted associations (green edges) or negative regularized adjusted associations (red edges). The thickness and color saturation of an edge denotes its weight (the strength of the association between two nodes). CSI: Central Sensitization Inventory; EQ5DL: EuroQol-5D questionnaire; HADS, Hospital Anxiety and Depression Scale; PCS: Pain Catastrophizing Scale; PSQI: Pittsburg Sleeping Quality Index; S-LANSS, self-reported version of the Leeds Assessment of Neuropathic Symptoms and Signs; TSK-11: Tampa Scale for Kinesiophobia.

Figure 1 graphs the network in the sample of COVID-19 survivors with post-COVID pain. Different associations between sensory-related and psychological/cognitive variables were observed. PainDETECT was associated with S-LANSS (ρ: 0.388) and CSI (ρ: 0.207). In addition, CSI was associated with HADS-A (ρ: 0.269), TSK-11 (ρ: 0.165) and female sex (ρ: 0.413). As expected, HADS-A was associated with HADS-D (ρ: 0.598) and TSK-11 with PCS (ρ: 0.405). The only negative association was between sleep quality and EQ-5D-5L (ρ: −0.162).

**Figure 2:**
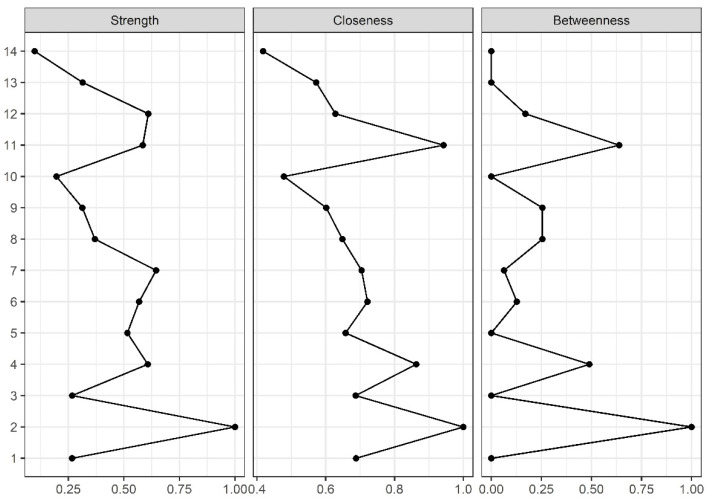
Centrality measures of Strength, Closeness, and Betweenness of each of the 14 nodes in the network. Centrality value of 1 indicates maximal importance, and 0 indicates no importance. The number of the variables are related to the network in Figure 1.

Gender (node 2) was the node with the highest strength, closeness, and betweenness centrality scores (Figure 2). In addition, CSI (node 11) was the node with the second highest closeness and betweenness centralities, whereas HADS-D (node 7) was the node with the second highest strength centrality (Figure 2).

**Figure 3:**
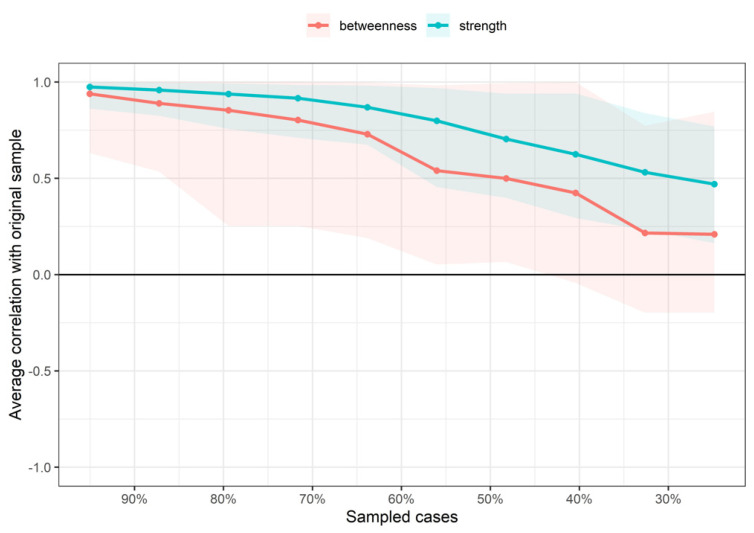
Average correlations between centrality indices of networks sampled with persons dropped and networks built on the entire input dataset, at all follow-up time points. Lines indicate the means and areas indicate the range from the 2.5th quantile to the 97.5th quantile.

The betweenness and closeness measures of the network were extremely unstable at CS_cor=0.7_ = 0.001 and CS_cor=0.7_ = 0.001, respectively. The strength centrality measure was found to be relatively stable with CS_cor=0.7_ = 0.28 (Figure 3).

## 4. Discussion

The is the first study applying a network analysis to phenotype post-COVID pain. The identified network supports a model where sensitization-associated symptoms, neuropathic phenotype, and cognitive/psychological aspects are significantly connected. The results support that post-COVID pain resembles a nociplastic pain condition where musculoskeletal and neuropathic pain processes are present at the same time. Current findings agree with the notion that discrimination between nociceptive, neuropathic, and nociplastic pain represent a current challenge and that nociplastic pain is a mechanistic term representing a continuum between musculoskeletal and neuropathic pain [30].

### 4.1. Importance of Neuropathic and Sensitization Mechanisms

The network identified two main features of post-COVID pain, a neuropathic pain component as expressed by the association between PainDETECT and S-LANSS questionnaires, and a sensitization-associated pain component as expressed by the associations with CSI. In fact, the neuropathic pain component (e.g., PainDETECT) was associated with sensitization-associated symptomatology (e.g., CSI). The fact that sensitization plays a relevant role in post-COVID pain has been previously suggested by Goudman et al. [11] using the CSI. However, it should be considered that the exclusive use of CSI for inferring sensitization in people with chronic pain is limited because it overlaps with psychological construct, and because a self-reported tool cannot capture the complexity of a central nervous system impairment such as sensitization [31]. In fact, the network also revealed an association between CSI and HADS-A supporting this overlapping between sensitization-associated symptoms and anxiety. Similarly, the association of CSI with kinesiophobia levels also supports that these PROMs overlap in cognitive/emotional/psychological aspects [20] and different constructs of sensitization, e.g., quantitative sensory testing, should be used to further confirm the presence of altered nociceptive processing in post-COVID pain.

The presence of sensitization-associated symptoms is mostly assumed to musculoskeletal pain conditions. The main finding revealed by the network is a significant association of sensitization-associated symptoms with the neuropathic pain component. This association supports that the neuropathic component is an important phenotype of post-COVID pain, at least in 20–25% of the patients [6]. Additionally, the fact that PainDETECT and S-LANSS were associated is an expected finding since both are PROMs used for evaluating neuropathic pain. A strong association between these two questionnaires has been previously found in patients with knee osteoarthritis [32]. Current and previous results suggest good convergent validity between both S-LANSS and PainDETECT; however, it is possible that these PROMs, although both evaluate the presence of neuropathic pain features, assess different aspects of the neuropathic pain spectrum.

### 4.2. Importance of Function

Other associations identified in the network, e.g., between kinesiophobia and pain catastrophism, increase evidence about the relevance of cognitive factors in post-COVID pain. This association has been previously found in musculoskeletal pain conditions such as plantar heel pain [33] or patellofemoral pain [34]. The presence of these maladaptive behaviours can promote chronification and persistence of pain [35]. Finally, the last association identified in the network was between the quality of sleep and health-related quality of life. Evidence supports that sleep problems are highly prevalent post-COVID associated symptoms [36]. Our network indicates that for improving health-related quality of life in COVID-19 survivors with post-COVID pain, proper management of sleep quality is needed.

### 4.3. Importance of Gender

The network also revealed that gender was the edge with the strongest weights (i.e., meaning that this node influences others or is influenced by others directly), supporting that management of post-COVID pain should considered sex differences. The relevance of female sex is also supported by the association between sensitization-associated symptoms (e.g., CSI) and gender. In such a scenario, the results from the current network suggest that if clinicians want to influence post-COVID pain, the best form to focus treatment on would be to specifically consider sex. That female sex is a risk factor for developing post-COVID symptoms in general, and also post-COVID pain, is supported in the literature [37]. This could be related to the fact that women experience musculoskeletal pain more frequently than men [38], and, accordingly, post-COVID pain should receive particular attention in females.

### 4.4. Clinical Applications

The associations identified in the current network have several implications for clinical practice. For instance, the presence of sensitization-associated symptoms would support why exercise, which effects are mainly mediated by the central nervous system, could be a therapeutic strategy to be applied in people with long COVI D [39]. Nevertheless, it is important to consider that sensitization is associated with poor clinical outcomes to conservative treatment in musculoskeletal pain conditions [40]. In fact, it is discussed that underlying pain mechanisms of each condition must be considered to optimize exercise prescription in individuals with nociplastic pain predominance [41]. Accordingly, the associations observed in this network should be individually identified on each patient with long COVID for prescription of personalized exercise programs. This clinical rational increases its relevance in COVID-19 survivors with post-COVID pain but also in those patients with other post-COVID symptoms such as fatigue or dyspnea or those with autonomic disturbances [42], since the exercise-induced response in these patients could be different than in other conditions. The results from our network further reinforce the proposal that management of long COVID should include multimodal therapeutic approaches targeting sensitization and neuropathic mechanisms (i.e., neuro-modulatory pain approaches such as exercise, pain education, or physical therapy) but also psychological/cognitive aspects (i.e., cognitive behavior, copying strategies) as well as sleep management strategies.

### 4.5. Limitations

The most relevant limitation associated with the network approach is that conditional independence relationships, as encoded by the edge weights in the networks, cannot be a source of confirmatory causal inference, but may provide indicative potential causal pathways. In other words, the network is supported if biological plausibility between those connected variables exists. Importantly, this assumption is supported in the relationships identified in our network. Nevertheless, the direction of the association, although it can be proposed based on current knowledge, cannot be confirmed without longitudinal designs. Despite this potential limitation, current findings provide hypotheses on underlying phenotypes of post-COVID pain and therapeutic targets for future clinical trials in this population.

## 5. Conclusions

The results of this study, by using a network analysis approach, revealed that post-COVID pain is phenotyping by complex associations between sensitization-associated symptoms, neuropathic symptomatology, and psychological/emotional aspects. Further, post-COVID pain was found to be gender dependent since female sex plays a relevant role. The associations identified in the current study further support that treatment of patients with long COVID should be include a multidisciplinary approach as recommended for managing complex chronic pain conditions.

## Figures and Tables

**Table 1:** Values (mean ± standard deviation) of demographic, quality of life, sensory, psychological and clinical variables of the total sample (*n* = 141).

Variable	
Age (years)	57.3 ± 11.7
Gender (male, *n*; %)	66; 46.8
Weight (kg)	81.8 ± 17.0
Height (cm)	1.65 ± 0.10
Post-COVID Duration (months)	18.8 ± 1.8
HADS-A (0–21)	5.2 ± 4.2
HADS-D (0–21)	4.9 ± 4.3
PSQI (0–21)	8.0 ± 4.2
PainDETECT (−1 to 38)	7.0 ± 6.2
S-LANSS (0–24)	7.5 ± 8.5
CSI (0–100)	33.9 ± 17.2
PCS (0–52)	12.3 ± 12.0
TSK-11 (0–44)	24.0 ± 8.6
EuroQol-5D-5L (0–1)	0.8 ± 0.2

CSI: Central Sensitization Inventory; EQ5DL: EuroQol-5D questionnaire; HADS, Hospital Anxiety and Depression Scale; PCS: Pain Catastrophizing Scale; PSQI: Pittsburg Sleeping Quality Index; S-LANSS, self-reported version of the Leeds Assessment of Neuropathic Symptoms and Signs; TSK-11: Tampa Scale for Kinesiophobia.

## Data Availability

All data derived from this study are presented in the text.
